# The impact of different GFR estimating equations on the prevalence of CKD and risk groups in a Southeast Asian cohort using the new KDIGO guidelines

**DOI:** 10.1186/1471-2369-13-1

**Published:** 2012-01-06

**Authors:** Chagriya Kitiyakara, Sukit Yamwong, Prin Vathesatogkit, Anchalee Chittamma, Sayan Cheepudomwit, Somlak Vanavanan, Bunlue Hengprasith, Piyamitr Sritara

**Affiliations:** 1 Department of Medicine, Faculty of Medicine, Ramathibodi Hospital, Mahidol University, Bangkok10110, Thailand; 2 Division of Clinical Chemistry, Department of Pathology, Faculty of Medicine, Ramathibodi Hospital, Mahidol University, Bangkok 10110, Thailand; 3 Medical and Health Office, Electricity Generating Authority of Thailand, Bangkruay, Nonthaburi 11130, Thailand

**Keywords:** EGAT, glomerular filtration rate, renal failure, epidemiology, classification, kidney, Thai

## Abstract

**Background:**

Recently, the Kidney Disease: Improving Global Outcomes (KDIGO) group recommended that patients with CKD should be assigned to stages and composite relative risk groups according to GFR (G) and proteinuria (A) criteria. Asians have among the highest rates of ESRD in the world, but establishing the prevalence and prognosis CKD is a problem for Asian populations since there is no consensus on the best GFR estimating (eGFR) equation. We studied the effects of the choice of new Asian and Caucasian eGFR equations on CKD prevalence, stage distribution, and risk categorization using the new KDIGO classification.

**Methods:**

The prevalence of CKD and composite relative risk groups defined by eGFR from with Chronic Kidney Disease-Epidemiology Collaboration (CKD-EPI); standard (S) or Chinese(C) MDRD; Japanese CKD-EPI (J-EPI), Thai GFR (T-GFR) equations were compared in a Thai cohort (n = 5526)

**Results:**

There was a 7 fold difference in CKD_3-5 _prevalence between J-EPI and the other Asian eGFR formulae. CKD_3-5 _prevalence with S-MDRD and CKD-EPI were 2 - 3 folds higher than T-GFR or C-MDRD. The concordance with CKD-EPI to diagnose CKD_3-5 _was over 90% for T-GFR or C-MDRD, but they only assigned the same CKD stage in 50% of the time. The choice of equation also caused large variations in each composite risk groups especially those with mildly increased risks. Different equations can lead to a reversal of male: female ratios. The variability of different equations is most apparent in older subjects. Stage G3aA1 increased with age and accounted for a large proportion of the differences in CKD_3-5 _between CKD-EPI, S-MDRD and C-MDRD.

**Conclusions:**

CKD prevalence, sex ratios, and KDIGO composite risk groupings varied widely depending on the equation used. More studies are needed to define the best equation for Asian populations.

## Background

Chronic kidney disease (CKD) increases the risks of cardiovascular disease and ESRD progressively according to the severity of CKD [1]. In 2002, the Kidney Disease Outcomes Quality Initiative (KDOQI) organization published a guideline for diagnosis and classification of CKD into five stages according to severity using glomerular filtration rate (GFR) as the main criteria [2]. The presence of proteinuria was mandatory for stages 1 and 2 (GFR > 60 ml/min/1.73 m^2^). Patients with stage 3 (GFR 30 to < 60) or higher were regarded classified as CKD without requirement for the presence of proteinuria. Recent studies have shown that individuals with GFR 45 ml/min/1.73 m^2 ^or less had increased risks compared to those with higher GFR [1,3-5]. The presence of proteinuria also increased cardio-renal events significantly [1]. Therefore, in 2009, KDIGO (Kidney Disease: Improving Global Outcomes) group recommended that individuals should be classified according to proteinuria stages as well as by GFR stages [1]. The diagnostic criteria for CKD remained unchanged, but stage 3 should be divided into 2 substages: 3a (GFR 45 to < 60), and 3b (GFR 30to < 45). In addition, clinicians and researchers were advised to use a 'heat map' generated by composite rankings of relative risks to categorize patients.

The numbers of patients in Asia including those in Thailand with ESRD has been rising steadily over the last decade [6,7]. Correct identification and staging of CKD is essential to target care to patients with highest risks and for planning of future healthcare policies. The pooled analyses used to develop the new KDIGO guidelines employed the standard MDRD (S-MDRD) equation to estimate GFR [3-5]. The S-MDRD equation was designed from US white and black patients with CKD [8]. It is well recognized that equations developed in one CKD population may be inaccurate when applied to another ethnic population or to those without known CKD. Currently, there is no universal equation for Asian subjects. MDRD based-equations have been developed in Japanese and Chinese populations [9,10], but they produced correction coefficients in opposite directions to one another. S-MDRD may lead to overestimate of GFR especially among those with higher GFR [11]. Chronic Kidney Disease-Epidemiology Collaboration (CKD-EPI) equation, was developed from subjects with and without kidney diseases, and therefore, may be preferred for studying GFR in a largely normal population sample [12]. More recently, a Japanese version for CKD-EPI (J-EPI) was shown to be more accurate compared to the Japanese MDRD equation in the Japanese population [13]. Finally, a Thai GFR equation has also been developed in a Thai CKD population [14].

It is the aim of KDIGO to establish global guidelines for CKD [1]. However, at present, it is unclear which equation(s) should be used in Asian populations to classify CKD. In addition, there is still limited data on the prevalence of CKD class 3 subdivisions, and the numbers in different risk categories in Asian populations. The prevalence of the subdivisions and each risk category will depend on the equation used to estimate GFR. In this study, we investigated the impact of different GFR equations developed in Caucasian and other Asian populations on GFR, and the prevalence of CKD in a Thai cohort using the new KDIGO guidelines.

## Methods

### Study participants

During 1997-1998, 6152 employees of the Electricity Generating Authority of Thailand (EGAT) were invited to participate in a health survey [15,16]. All participants completed a self-administered questionnaire and underwent a physical examination. Blood samples were drawn after 12 hours overnight fast. The study was approved by the ethical committees of EGAT and Ramathibodi Hospital. Written informed consent was obtained.

### Laboratory measurements

Serum creatinine (sCr) was measured using a IDMS-standardized enzymatic assay on the Vitros 350 analyzer (Ortho-Clinical Diagnostics, USA), which has been shown to produce similar results to the Roche assay used in the MDRD study [17-19]. Details of the calibration procedure have been published [20]. Calibration was performed using two levels (low; 0.753 mg/dL ± 0.021 and high; 3.916 mg/dL ± 0.083) of IDMS-Standard Reference Material (SRM) 967. Mean concentrations (coefficients of variation) for the two levels of SRM 967 were 0.749 mg/dL (1.64%), and 3.898 mg/dL (0.41%), respectively. Stable quality control was maintained throughout using the manufacturer's quality control materials. Urine protein was detected by urinalysis reagent strip (Bayer, Indiana, USA).

### Estimated GFR (eGFR) Equations

SCr (mg/dl) was used to determine eGFR (ml/min/1.73 m^2^) according to different formulae:

a) Chronic Kidney Disease-Epidemiology Collaboration (CKD-EPI) = 141 × min(sCr/κ, 1)α × max(sCr/κ, 1)-1.209 × 0.993Age × 1.018 [if female] [12]

where κ is 0.7 for females and 0.9 for males, α is -0.329 for females and -0.411 for males, min indicates the minimum of sCr/κ or 1, and max indicates the maximum of sCr/κ or 1.

b) Standard MDRD (S-MDRD) = 175 × sCr ^-1.154 ^× Age ^-0.203 ^× 0.742 [if female] [17]

c) Chinese MDRD (C-MDRD) = 175 × sCr ^-1.154 ^× Age ^- 0.203 ^× 1.233 × 0.742 [if female] [9,21]

d) Japanese CKD-EPI (J-EPI) = 0.813 × CKD-EPI [13]

e) Thai GFR (T-GFR) = 375.5 × sCr ^0.848 ^× Age^-0.364 ^× 0.712 [ if female] [14]

### CKD staging

Subjects were divided into 5 stages by eGFR (G1-5) and 3 stages of albuminuria (A1-3) according to the 2009 KDIGO guidelines [1]. Because we did not have albuminuria data, dipstick protein was used instead as follows: A1proteinuria dipstick negative or trace, A2 1or 2+, A3 protein 3+ or more. CKD prevalence was assessed using different equations. CKD_ALL _represents CKD stages 1 to 5. CKD _3-5 _represents eGFR < 60 ml/min/1.73 m2 regardless of proteinuria data. One of the concerns was the high numbers of patients with CKD _3-5 _in the older age groups, in particular, those patients with stage 3a without proteinuria (G3A1) [1]. Therefore, we also evaluated the prevalence of G3A1 in different age groups.

### Concordance

*CKD_3-5 _status concordance *was assessed in pairs between the three Asian equations versus CKD-EPI or S-MDRD, and also between T-GFR versus the other Asian equations. Concordant subjects included subjects who fulfilled the criteria for CKD*_3-5 _*by both tested equations, and subjects who did not fulfill the criteria for CKD*_3-5 _*by both equations. The *CKD_3-5 _status concordance *was expressed as a percentage of *all *subjects.

*CKD_ALL _stage concordance *was assessed in pairs between the three Asian equations versus CKD-EPI or S-MDRD, and also between T-GFR versus the other Asian equations. Subjects with no CKD (stage G1A1 or G2A1) by both tested equations were excluded from the analysis. Concordant subjects were those who were assigned the same CKD stage by both pairs of the test equations. This was expressed as a percentage of those with CKD_ALL _by either one of the pair using the KDIGO classification [1] in which stage 3a and stage 3b were considered as separate stages or by the KDOQI [2] classification in which stage 3 was considered as one stage.

### Composite ranking of relative risks

The composite rankings for relative risks by GFR and proteinuria was calculated based on 2009 KDIGO recommendations using different equations as follows: Risk Category No CKD (group 1-8) stages G1A1, G2A1; Mild (group 9-14) stages G1A2, G2A2, G3aA1; Moderate (group 15-21) stages G1A3, G2A3, G3aA2, G3bA1; Severe (group ≥ 22) stages G3aA3, G3bA2-3, all G4 and G5[1].

### Statistical analyses

Continuous data is reported as mean (95% confidence intervals). Categorical data is presented as frequency and percentages. Continuous variables are compared using Student's t test or Wilcoxon rank sum test as appropriate. Categorical variable were compared using chi square test. All tests were two tailed, and a p-value less than 0.05 is considered statistically significant. All statistical analyses were performed using SPSS software (SPSS Version 18; SPSS Inc, Chicago, IL).

## Results

### Study participants

A total of 5966 volunteered to be screened. Of these, 5526 subjects had complete data for analysis (table 1). Almost all subjects were Thais or Thai-Chinese. About 75% of subjects were males, 11.6% had diabetes and 9.9% had proteinuria. Men were older and more likely to have higher BMI, blood pressure, DM, and hypertension.

**Table 1:** Participant Characteristics

	All (n = 5526)	Males (n = 4121)	Females (n = 1405)	P
Age (years)	48.4 (48.2-48.6)	48.7 (48-5-48.9)	47.5 (47.1-47,8)	< .001
Weight (kg)	64.8 (64.5-65.1)	67.3 (67.0-67.6)	57.5 (57.0-58.0)	< .001
BMI (kg/m^2^)	24.25 (24.16-24.34)	24.35 (24.25-24.45)	23.95 (23.76-24.15)	< .001
SBP (mm Hg)	130.0 (129.4-130.5)	133.2 (132.6-133.8)	120.6 (119.6-121.6)	< .001
DBP(mmHg)	79.2 (78.8-79.5)	81.1 (80.7-81.4)	73.7 (73.1-74.3)	< .001
S creatinine (mg/dl)	1.08 (1.07-1.09)	1.15 (1.14-1.17)	0.86 (0.85-0.87)	< .001
Proteinuria (%)	9.9%	10.3%	8.8%	NS
DM (%)	11.6	12.5	9.0	< .001
Hypertensive (%)	36.3	41.1	22.3	< .001

BMI, Body mass index; SBP systolic blood pressure; DBP diastolic blood pressure, DM diabetes mellitus

### All patients

#### Glomerular Filtration rate

GFR obtained by different equations were different to one another (p < 0.001). C-MDRD and T-GFR produced higher mean GFR whereas J-EPI produced much lower GFR estimates (table 2). S-MDRD and CKD-EPI produced intermediate values. The maximum difference in mean GFR was over 30 ml/min/1.73 m^2 ^ranging from 89.7 for C-MDRD to 64.6 with J-EPI.

**Table 2:** GFR according to different equations (n = 5526)

Equation (N)	All (5526)	Males (4121)	Females (1405)
**S-MDRD**	72.6 (72.1-73.0)	71.7 (71.1-72.2)	75.2 (74.2-76.2)
**CKD-EPI**	79.4 (78.9-79.8)	77.9 (77.3-78.3)	84.0 (83.0-84.9)
**J-EPI**	64.6 (64.2-65.0)	63.4 (62.9-63.8)	68.3 (67.7-69.1)
**C-MDRD**	89.6 (89.1-90.2)	88.6 (87.9-89.2)	92.9 (91.7-94.1)
**T-GFR**	82.4 (83.3-82.9)	84.5 (84.0-85.0)	78.0 (77.2-78.8)

Data shown as mean (95% CI) in ml/min/1,73 m^2^.

eGFR by using one equation was different from eGFR by

all other equations (p < 0.001) for all, males and females

CKD-EPI, Chronic Kidney Disease-Epidemiology Collaboration

S-MDRD, standard MDRD; C-MDRD, Chinese MDRD; J-EPI, Japanese CKD-EPI; T-GFR, Thai GFR.

#### CKD prevalence

The prevalence of CKD_ALL _between various equations was different (p < 0.001) except for between TGF and C-MDRD. The prevalence of CKD_ALL _for J-EPI was 3 folds higher compared to T-GFR or C-MDRD (table 3). The prevalence of CKD_ALL _was intermediate for the 2 Caucasian equations. CKD_ALL _with S-MDRD was nearly two times higher than with T-GFR. The prevalence of CKD_ALL _with CKD-EPI was about 5% higher than with T-GFR.

**Table 3:** GFR and proteinuria stages, and CKD prevalence by different equations according to 2009 KDIGO classification

	G1			G2			G3a			G3b			G4			G5			CKD ALL	CKD 3-5
**CKD-EPI**	27.5			60.4			9.2			2.3			0.6			0.1			19.7^a^	12.1^a^
	25.5	1.8	0.2	54.8	5.1	0.5	7.6	1.5	0.1	1.7	0.5	0.1	0.5	0.1	0.0	0.0	0.0	0.0		

**S-MDRD**	11.2			67.5			16.8			2.7			0.6			0.1			27.1^a^	20.2^a^
	11.1	1.0	0.1	61.8	5.2	0.5	14.6	2.0	0.2	2.0	0.6	0.1	0.5	0.1	0.0	0.0	0.0	0.0		

**J-EPI**	3.2			58.9			30.7			5.8			1.3			0.1			43.0^a^	37.9^a^
	2.9	0.2	0.1	54.1	4.4	0.4	27.4	3.0	0.3	4.7	1.0	0.1	1.0	0.2	0.1	0.1	0.0	0.0		

**C-MDRD**	45.6			48.9			4.0			1.3			0.2			0.05			14.2^b^	5.5^b^
	41.7	3.5	0.4	44.1	4.4	0.4	3.1	0.8	0.1	1.0	0.2	0.1	0.2	0.0	0.0	0.0	0.0	0.0		

**T-GFR**	29.2			65.5			4.3			1.1			0.1			0.04			14.0	5.4
	27.1	1.8	0.3	58.9	6.0	0.6	3.2	1.0	0.1	0.9	0.2	0.0	0.1	0.0	0.0	0.0	0.0	0.0		

Data shown as percentages of all subjects (n-5526) for each GFR stage (G1-5) and for each proteinuria subdivision

(represented as 3 subdivisions of each G stage from A1 to A2 to A3 from left to right). CKD_ALL _CKD from stage 1 to 5, CKD3-5 CKD stages 3 to 5

a CKD prevalence different compared to all other equations (p < 0.001)

b CKD prevalence different to other equations (p < 0.001) except with T-GFR

CKD-EPI, Chronic Kidney Disease-Epidemiology Collaboration; S-MDRD, standard MDRD; C-MDRD, Chinese MDRD; J-EPI, Japanese CKD-EPI; T-GFR, Thai GFR

The prevalence of CKD_3-5 _between various equations was different (p < 0.001) except for between TGF and C-MDRD. J-EPI produced 7 folds higher estimates than T-GFR and C-MDRD. The 2 Caucasian formulae produced intermediate results, but CKD_3-5 _was still 2 and 3 folds higher than T-GFR. The prevalence of CKD stages G1 and G2 were higher for T-GFR and C-MDRD, whereas stage G3 was much higher for J-EPI. Compared to others, a marked increase in stage G4 was also found for J-EPI. Differences in G3aA1 accounted for a large portion of the differences between equations.

#### CKD _3-5 _status concordance

**Figure 1:**
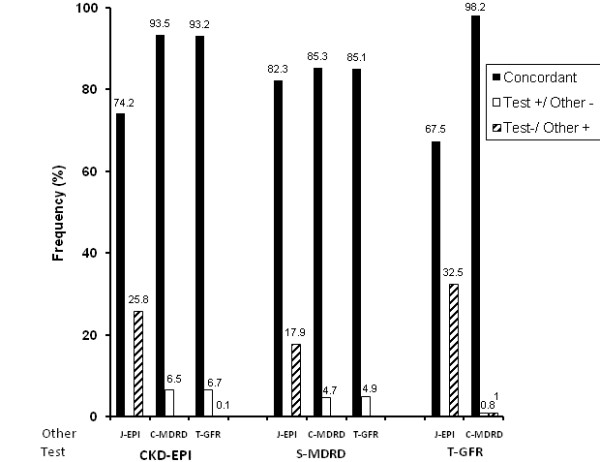
**CKD _3-5 _status concordance**. Concordant subjects include subjects with (+/+) or without (-/-) CKD by both pairs of test vs. other equation. Data expressed as percentage of all subjects (n = 5526). Discordant subjects are represented by +/- or -/+. CKD-EPI. CKD-EPI, Chronic Kidney Disease-Epidemiology Collaboration; S-MDRD, standard MDRD; C-MDRD, Chinese MDRD, J-EPI Japanese CKD-EPI, T-GFR Thai GFR.

The concordance (Figure 1) with CKD-EPI was over 90% for T-GFR and C-MDRD with both the Asian equations under-diagnosing CKD _3-5 _compared to CKD-EPI. On the other hand, the concordance of CKD-EPI with J-EPI was only 74.2% with J-EPI over-diagnosing CKD_3-5 _compared to CKD-EPI. The concordance for all Asian equations and S-MDRD were only moderate and slightly over 80%. C-MDRD and T-GFR tends to under-diagnose CKD_3-5 _whereas J-EPI tends to over-diagnose CKD _3-5_. The concordance between T-GFR and C-MDRD was excellent, whereas it was extremely poor with J-EPI.

#### CKD _ALL _stage concordance

The stage concordance was higher with the 2002 KDOQI criteria than with the 2009 KDIGO criteria. CKD_ALL _stage concordance with CKD-EPI was only 50-60% for T-GF and C-MDRD, but only 15-30% for J-EPI. In contrast, stage concordance with S-MDRD was 45-55% for J-EPI and only 26-39% for C-MDRD or T-GFR. The stage concordance with T-GFR was very poor for J-EPI, but over 70% for C-MDRD.

#### Composite ranking of relative risks

**Figure 2:**
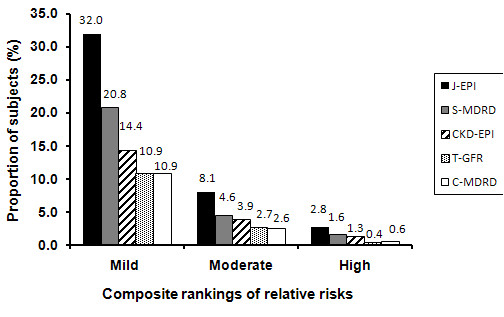
**Proportion of subjects according to KDIGO 2009 risk categories**. Mild (group 9-14): stages 1A2, 2A2, 3aA1; Moderate (group 15-21) stages 1A3, 2A3, 3aA2, 3bA1; Severe (group ≥ 22): stages 3aA3, 3bA2-3, All 4 and 5. Data expressed as percentages of all subjects (n = 5526). CKD-EPI. CKD-EPI, Chronic Kidney Disease-Epidemiology Collaboration; S-MDRD, standard MDRD; C-MDRD, Chinese MDRD, J-EPI Japanese CKD-EPI, T-GFR Thai GFR.

For all equations, the numbers of subjects in each risk category decreased with increasing severity of risks (Figure 2). The absolute numbers decreased approximately 4-5 folds from mild to moderate and by the same proportion from moderate to severe. The numbers varied strikingly according the equation used, but followed the same ranking pattern across severity groups. The absolute difference between equations varied most in the mild category which ranged from 32% in J-EPI to 10.9% in T-GFR or C-MDRD and were 20.8% and 14.0% for S-MDR and CKD-EPI respectively.

### Gender

**Figure 3:**
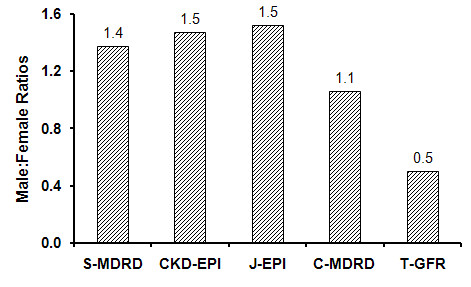
**Male: female ratios for CKD _3-5 _prevalence by different equations**. (n = 5526). CKD-EPI, Chronic Kidney Disease-Epidemiology Collaboration; S-MDRD, standard MDRD; C-MDRD, Chinese MDRD; J-EPI, Japanese CKD-EPI; T-GFR, Thai GFR.

Mean GFR was higher in females than in males for all equations except T-GFR (Table 2). The mean female male difference ranged from - 6.5 ml/min/1.73 m^2 ^for T-GFR to + 6.1 ml/min/1.73 m^2 ^for CKD-EPI. In both males and females, C-MDRD produced the highest GFR estimates whereas J-EPI produced the lowest GFR estimates. Different equations had marked effects on the prevalence of male to female ratios of CKD _3-5 _(Figure 3) which ranged from 0.5 for T-GFR to 1.5 for CKD-EPI.

### Age

Subjects were divided into 3 age groups: I (Age 20-39), n = 908 (15.2%); II (Age 40-59), n = 4184 (75.6%) and III (Age ≥ 60), n = 434 (7.9%). eGFR decreased (data not shown), and CKD_3-5 _increased with age for all equations. The absolute numbers varied widely, although the relative order was similar across all age groups. In Age group I, the prevalence of CKD _3-5 _varied by over 100 folds. In Age group III, there was a marked difference in CKD prevalence ranging from 14.7 to 63.1% for C-MDRD to J-EPI, respectively. The prevalence of CKD for all age groups was intermediate for the S-MDRD or CKD-EPI, but the differences in CKD _3-5 _in the oldest group was as high as 10 to 15% compared to T-GFR or C-MDRD.

The prevalence of both G3aA1 and higher stage CKD _3-5 _(CKD _3aA2-5_) increased with age for all equations. The ratios of G3aA1 to CKD _3aA2-5_varied with different equations, and decreased with age. G3aA1 accounted for nearly all the patients with CKD _3-5 _in Age group I, between 57 to 72% in Age group II, and between 35 to 57% in age group III. The prevalence of G3A1 and CKD _3aA2-5 _was much higher for J-EPI for all age groups. There were 2 to 5 fold variations in 3aA1 in the oldest age group. The prevalence was as high as 20.7% with S-MDRD equation and only 7-8% for C-MDRD or T-GFR. In Age group III, much of the differences in CKD _3-5 _between S-MDRD, CKD-EPI, T-GFR could be accounted for by differences in G3A1 since the prevalence of CKD _3aA2-5 _were similar.

## Discussions

Correct estimation of GFR is essential to diagnose and stage CKD, to assess individual risks, and to allowing comparisons between different populations in epidemiologic studies. The KDIGO analyses included some patients from China and Japan, but most of the data available for analysis is of mainly Caucasian or US black subjects with eGFR calculated using S-MDRD formula [1]. This study found that GFR estimates, CKD prevalence, proportions with increased risks, and gender ratios in this Asian cohort varied widely depending on the equation used. To our knowledge this is the first study to evaluate the use of J-EPI and T-GFR equations on the prevalence of CKD, and the concordance of these new Asian formulae on CKD staging using the new KDIGO guidelines.

In this study, CKD _3-5 _prevalence was as high as 37.9% with J-EPI whereas the prevalence was only 5-6% with T-GFR or C-MDRD, 20.2% with S-MDRD and 12.1% with CKD-EPI. The importance of choice of eGFR equations on CKD prevalence have been shown previously in studies from the US and Europe [8,11,22]. In these studies, changing from S-MDRD to CKD-EPI resulted in lower CKD prevalence estimates. In US white and black subjects, CKD-EPI has emerged as the preferred equation for population studies as it has been shown to be more accurate in CKD stage classification than the MDRD study equation [11,12]. At present, it remains unclear which is the best formula for Asian subjects. CKD-EPI has recently been shown not to provide consistent prediction of true GFR when applied to Asian subjects [23].

In diagnosing moderate CKD (eGFR < 60) in a general population, the concordance of T-GFR or C-MDRD with CKD-EPI was over 90% compared to only 80% with S-MDRD. In assigning stage for all subjects with CKD (from stages 1 to 5), the concordance of T-GFR or C-MDTRD equations was only about 50-60% with CKD-EPI, and less than 40% with S-MDRD. The concordance between T-GFR and C-MDRD was high, but both had very poor concordance with J-EPI. Therefore, it appears that T-GFR, C-MDRD and CKD-EPI may produce comparable results in identifying individuals with moderate CKD, but the CKD stage assigned, if all stages of CKD were assessed, may be different in half the time.

Using the 2009 KDIGO guidelines to subdivide stage 3, we showed that the CKD _ALL_, stage concordance between the Asian and Caucasian equations decreased by about 5-9% when compared to the KDOQI classification in which stage 3 was considered as one stage. KDIGO also recommended the use of composite rankings of relative risks according to GFR and proteinuria stages to assess individuals. In our analysis, the choice of equation affected the numbers at risk quite markedly especially those with mildly increased risks. Taken together this data suggests that the choice of eGFR equation has very considerable impact in diagnosing CKD, CKD staging, individual risk assessment.

This study demonstrated that changing eGFR equations could affect CKD gender ratios quite dramatically. Similar to our study, the male to female CKD ratio among US NHANES subjects increased slightly when the GFR equation was changed from S-MDRD to CKD-EPI [12]. However, the largest difference in our study was found when T-GFR was used, since this led to a reversal in the male to female ratio even when compared to C-MDRD. This finding, however, is not consistent with the slightly higher male to female ratios among those receiving renal replacement therapy in Thailand and will require further analysis.

We showed that both stage G3aA1 and higher stages of CKD increased with age, but the absolute numbers and relative proportions depend on the equations used. Young and middle age subjects with stage 3aA1 have been shown to be at increased risks of renal events, cardiac deaths and all cause mortality [4,24]. Among older patients, similar increases in risk for renal events were found, but the effects on cardiovascular deaths were less consistent. In the oldest age group, nearly all the differences CKD 3-5 prevalence between S-MDRD, CKD-EPI and C-MDRD could be accounted for by differences in stage 3aA1. Clearly the magnitude of attributed risks for developing cardio-renal complications for this stage will depend on the equation used to stage CKD.

Differences between the Asian equations may be due to differences in GFR measurement methods, creatinine calibration or true differences in the study populations [25,26]. J-EPI is now the equation of choice in the Japanese population [13]. Muscle mass is a major determinant in the relationship between serum creatinine and true GFR [25]. Muscle mass is lower in Japanese compared to American subjects [26,27]. This is consistent with a coefficient of < 1 for the Japanese equation, but still cannot account for the very high prevalence of CKD when this equation is applied to other Asian populations. Indeed, when applied to the Thai population as in this study, the prevalence of CKD is far too high for J-EPI equation to be accurate.

This study employed IDMS-standardized enzymatic method to measure serum creatinine similar to S-MDRD, CKD-EPI, T-GFR and J-EPI. On the other hand, C-MDRD was derived from Jaffe method after calibration to the Cleveland clinic laboratory, which was used to develop the MDRD equation [9]. By applying a correction factor to adjust for standardized enzymatic creatinine and Cleveland clinic Jaffe creatinine, systematic differences between the two methods can be minimized [21]. Nonetheless, creatinine method differences could account for some differences between C-MDRD and other equations [25].

Methods to measure the reference GFR may contribute to the differences between equations [28,29]. Iothalamate clearance, used in the S-MDRD study, has been shown to overestimate GFR when compared to standard inulin clearance [30,31] and this could contribute to the lower Japanese correction coefficient. Plasma clearance of ^99m^Tc-DTPA was used as a reference GFR method in C-MDRD and T-GFR [9,14]. DTPA could overestimate GFR when compared to inulin clearance [31]. In addition, Chinese and Thai studies employed quite short clearance protocols which could further contribute to overestimation of GFR compared to inulin [28] The similarities for the T-GFR and C-MDRD equations may in part reflect their common use of DTPA as the reference method [9,14]. Slight differences in protocol may contribute to the different coefficients. Both Chinese, Japanese, and Thai studies included only CKD patients, and hence it is uncertain how well these formulae to can be applied to normal subjects.

The limitations of this study include the fact that proteinuria was defined by dipstick. Although previous studies have shown that dipstick proteinuria provide similar risk prediction as albumin excretion [1], the use of albumin to creatinine ratio would have allowed the inclusion of those with microalbuminuria, and lead to more accurate staging and risk assessment for our subjects. Secondly, this study was designed to collect cardiovascular risk factors, and hence there is limited data on subjects with known kidney diseases beyond the presence of diabetes and hypertension.

Asians have among the highest rates of ESRD in the world [6,7]. The Thai Renal Replacement Registry data showed the steep increase of renal replacement prevalence from 302.6 per million populations in 2006 to 496.9 in 2008. Nonetheless, accurate estimation of CKD prevalence remains a problem for Asian populations. The prevalence of CKD_3-5 _observed in our study using S-MDRD was fairly high, but is comparable to the 15% observed in other population surveys from younger subjects in Thailand, in which S-MDRD was used to classify CKD [20,32]. Such high rate of CKD is a concern. It is uncertain if the high rates reflect inappropriate application of the Caucasian equations to the Thai population or a genuine increase in CKD in our population. It remains unclear which equation should be used to classify CKD in Thai or other Asian subjects. Although T-GFR was developed in Thai CKD subjects, T-GFR equation may not be the ideal equation in our population since there may be a bias especially among those with lower GFR. For example, the creatinine value consistent with a GFR of 5 ml/min/1.73 m^2 ^in a 50 year old male would be 11 mg/dl and 30 mg/dl by S-MDRD and T-GFR studies [14]. Nonetheless, by reclassifying our patients with the T-GFR or C-MDRD will lead to a reduction of those with CKD _3-5 _by 2 to 3 folds with the impact greatest among the elderly group.

## Conclusions

This study showed that there is remarkable degree of variability in CKD prevalence and risk estimates when current equations developed in Caucasians and Asians were applied to the Thai population. The results of this study indicate that there is a need to develop a universal Asian equation using standardized creatinine, and valid common GFR reference method. The staging of CKD initiated by KDOQI, and subsequently refined in the 2009 KDIGO convention represents major advances in the field, but how best to apply such a staging system on a global basis will require further study. The use of the term 'disease' to describe asymptomatic laboratory condition may cause unnecessary concern in patients and clinicians. Identification of those truly at high risks would enable targeting of scarce resources to those with the greatest need. This is especially important in countries with limited resources such as many places in Asia, who may not have developed equations of their own.

## Competing interests

The authors declare that they have no competing interests.

## Authors' contributions

CK and SY are involved in data analysis and manuscript preparation. PV, SC, BH are involved in subject recruitment, database management. AC and SV are involved in laboratory standardization and measurements of serum creatinine. PR is the project leader and is responsible for co-ordination and funding of the project. All authors read and approved the final manuscript.

## Pre-publication history

The pre-publication history for this paper can be accessed here:

http://www.biomedcentral.com/1471-2369/13/1/prepub

## References

[B1] Levey AS de Jong PE Coresh J Nahas ME Astor BC Matsushita K Gansevoort RT Kasiske BL Eckardt KU The definition, classification and prognosis of chronic kidney disease: a KDIGO Controversies Conference report Kidney Int 2010 10.1038/ki.2010.483 21150873

[B2] K/DOQI clinical practice guidelines for chronic kidney disease: evaluation, classification, and stratification Am J Kidney Dis 2002 39 2 Suppl 1 S1 266 11904577

[B3] Astor BC Matsushita K Gansevoort RT van der Velde M Woodward M Levey AS Jong PE Coresh J de Jong PE El-Nahas M Lower estimated glomerular filtration rate and higher albuminuria are associated with mortality and end-stage renal disease. A collaborative meta-analysis of kidney disease population cohorts Kidney Int 2011 10.1038/ki.2010.550 PMC3917543 21289598

[B4] Gansevoort RT Matsushita K van der Velde M Astor BC Woodward M Levey AS Jong PE Coresh J de Jong PE El-Nahas M Lower estimated GFR and higher albuminuria are associated with adverse kidney outcomes in both general and high-risk populations. A collaborative meta-analysis of general and high-risk population cohorts Kidney Int 2011 10.1038/ki.2010.531 PMC3959732 21289597

[B5] Matsushita K van der Velde M Astor BC Woodward M Levey AS de Jong PE Coresh J Gansevoort RT Association of estimated glomerular filtration rate and albuminuria with all-cause and cardiovascular mortality in general population cohorts: a collaborative meta-analysis Lancet 2010 375 9731 2073 2081 20483451 10.1016/S0140-6736(10)60674-5 PMC3993088

[B6] Hossain MP Goyder EC Rigby JE El Nahas M CKD and poverty: a growing global challenge Am J Kidney Dis 2009 53 1 166 174 10.1053/j.ajkd.2007.10.047 19101400

[B7] Glassock RJ Winearls CG The global burden of chronic kidney disease: how valid are the estimates? Nephron Clin Pract 2008 110 c39 c46 10.1159/000151244 18689986

[B8] Levey AS Bosch JP Lewis JB A more accurate method to estimate glomerular filtration rate from serum creatinine: a new prediction equation. Modification of Diet in Renal Disease Study Group Ann Intern Med 1999 130 461 470 10075613 10.7326/0003-4819-130-6-199903160-00002

[B9] Ma YC Zuo L Chen JH Luo Q Yu XQ Li Y Xu JS Huang SM Wang LN Huang W Modified glomerular filtration rate estimating equation for Chinese patients with chronic kidney disease J Am Soc Nephrol 2006 17 10 2937 2944 10.1681/ASN.2006040368 16988059

[B10] Matsuo S Imai E Horio M Yasuda Y Tomita K Nitta K Yamagata K Tomino Y Yokoyama H Hishida A Revised equations for estimated GFR from serum creatinine in Japan Am J Kidney Dis 2009 53 6 982 992 10.1053/j.ajkd.2008.12.034 19339088

[B11] Levey AS Stevens LA Estimating GFR using the CKD Epidemiology Collaboration (CKD-EPI) creatinine equation: more accurate GFR estimates, lower CKD prevalence estimates, and better risk predictions Am J Kidney Dis 2010 55 4 622 627 10.1053/j.ajkd.2010.02.337 20338463 PMC2846308

[B12] Levey AS Stevens LA Schmid CH Zhang YL Castro AF Feldman HI Kusek JW Eggers P Van Lente F Greene T A new equation to estimate glomerular filtration rate Ann Intern Med 2009 150 9 604 612 19414839 10.7326/0003-4819-150-9-200905050-00006 PMC2763564

[B13] Horio M Imai E Yasuda Y Watanabe T Matsuo S Modification of the CKD epidemiology collaboration (CKD-EPI) equation for Japanese: accuracy and use for population estimates Am J Kidney Dis 2010 56 1 32 38 10.1053/j.ajkd.2010.02.344 20416999

[B14] Praditpornsilpa K Townamchai N Chawatanarat T Tiranathanagul K Katawatin P Susantitapong P Trakarnvanich T Kanjanabuch T Avihingsanon Y Tungsanga K The need for robust validation for MDRD-based glomerular filtration rate estimation in various CKD populations Nephrol Dial Transplant 2011 10.1093/ndt/gfq815 21357214

[B15] Sritara P Cheepudomwit S Chapman N Woodward M Kositchaiwat C Tunlayadechanont S Sura T Hengprasith B Tanphaichitr V Lochaya S Twelve-year changes in vascular risk factors and their associations with mortality in a cohort of 3499 Thais: the Electricity Generating Authority of Thailand Study Int J Epidemiol 2003 32 3 461 468 10.1093/ije/dyg105 12777437

[B16] Aekplakorn W Bunnag P Woodward M Sritara P Cheepudomwit S Yamwong S Yipintsoi T Rajatanavin R A risk score for predicting incident diabetes in the Thai population Diabetes Care 2006 29 8 1872 1877 10.2337/dc05-2141 16873795

[B17] Levey AS Coresh J Greene T Stevens LA Zhang YL Hendriksen S Kusek JW Van Lente F Using standardized serum creatinine values in the modification of diet in renal disease study equation for estimating glomerular filtration rate Ann Intern Med 2006 145 4 247 254 16908915 10.7326/0003-4819-145-4-200608150-00004

[B18] Hutayanon P Sarakarn P Buakhamsri A Boonsom W Yamwong S The effect of the public or private status of health care facility in acute coronary syndrome: data from Thai ACS Registry J Med Assoc Thai 2007 90 Suppl 1 98 108 18431892

[B19] Myers GL Miller WG Coresh J Fleming J Greenberg N Greene T Hostetter T Levey AS Panteghini M Welch M Recommendations for improving serum creatinine measurement: a report from the Laboratory Working Group of the National Kidney Disease Education Program Clin Chem 2006 52 1 5 18 10.1373/clinchem.2005.0525144 16332993

[B20] Ingsathit A Thakkinstian A Chaiprasert A Sangthawan P Gojaseni P Kiattisunthorn K Ongaiyooth L Vanavanan S Sirivongs D Thirakhupt P Prevalence and risk factors of chronic kidney disease in the Thai adult population: Thai SEEK study Nephrol Dial Transplant 2010 25 5 1567 1575 10.1093/ndt/gfp669 20037182

[B21] Levey AS Coresh J Greene T Expressing the Modification of Diet in Renal Disease Study equation for estimating glomerular filtration rate with standardized serum creatinine values Clin Chem 2007 53 766 772 10.1373/clinchem.2006.077180 17332152

[B22] Giavarina D Cruz DN Soffiati G Ronco C Comparison of estimated glomerular filtration rate (eGFR) using the MDRD and CKD-EPI equations for CKD screening in a large population Clin Nephrol 2010 74 5 358 363 20979944 10.5414/cnp74358

[B23] Stevens LA Claybon MA Schmid CH Chen J Horio M Imai E Nelson RG Van Deventer M Wang HY Zuo L Evaluation of the Chronic Kidney Disease Epidemiology Collaboration equation for estimating the glomerular filtration rate in multiple ethnicities Kidney Int 2011 79 5 555 562 10.1038/ki.2010.462 21107446 PMC4220293

[B24] van der Velde M Matsushita K Coresh J Astor BC Woodward M Levey A de Jong P Gansevoort RT Levey AS de Jong PE Lower estimated glomerular filtration rate and higher albuminuria are associated with all-cause and cardiovascular mortality. A collaborative meta-analysis of high-risk population cohorts Kidney Int 2011 10.1038/ki.2010.536 21307840

[B25] Rule AD Teo BW GFR estimation in Japan and China: what accounts for the difference? Am J Kidney Dis 2009 53 6 932 935 10.1053/j.ajkd.2009.02.011 19463761 PMC2687408

[B26] Sanada K Kuchiki T Miyachi M McGrath K Higuchi M Ebashi H Effects of age on ventilatory threshold and peak oxygen uptake normalised for regional skeletal muscle mass in Japanese men and women aged 20-80 years Eur J Appl Physiol 2007 99 5 475 483 10.1007/s00421-006-0375-6 17186296

[B27] Janssen I Heymsfield SB Wang ZM Ross R Skeletal muscle mass and distribution in 468 men and women aged 18-88 yr J Appl Physiol 2000 89 1 81 88 10904038 10.1152/jappl.2000.89.1.81

[B28] Agarwal R Bills JE Yigazu PM Abraham T Gizaw AB Light RP Bekele DM Tegegne GG Assessment of iothalamate plasma clearance: duration of study affects quality of GFR Clin J Am Soc Nephrol 2009 4 1 77 85 10.2215/CJN.03720708 19005012 PMC2615714

[B29] Michels WM Grootendorst DC Rozemeijer K Dekker FW Krediet RT Glomerular filtration rate measurements by 125I-iothalamate should be corrected for inaccurate urine collections with 131I-hippuran Clin Nephrol 2009 72 5 337 343 19863875 10.5414/cnp72337

[B30] Odlind B Hallgren R Sohtell M Lindstrom B Is 125I iothalamate an ideal marker for glomerular filtration? Kidney Int 1985 27 1 9 16 10.1038/ki.1985.3 3920429

[B31] Perrone RD Steinman TI Beck GJ Skibinski CI Royal HD Lawlor M Hunsicker LG Utility of radioisotopic filtration markers in chronic renal insufficiency: simultaneous comparison of 125I-iothalamate, 169Yb-DTPA, 99mTc-DTPA, and inulin. The Modification of Diet in Renal Disease Study Am J Kidney Dis 1990 16 3 224 235 2205098 10.1016/s0272-6386(12)81022-5

[B32] Perkovic V Cass A Patel AA Suriyawongpaisal P Barzi F Chadban S Macmahon S Neal B High prevalence of chronic kidney disease in Thailand Kidney Int 2008 73 4 473 479 10.1038/sj.ki.5002701 18059458

